# Case report: Ultrasound-guided intrauterine biopsy and RF ablation therapy for fetal posterior neck solid tumor: first successful report

**DOI:** 10.3389/fonc.2022.913694

**Published:** 2022-08-23

**Authors:** Shanshan Liu, Qiong Luo, Xiaofeng Fu, Minyan Wang, Qingguo Zou, Licheng Wang, Guangjuan Kan, Xing Si, Heqin Dong, Xiani Lan, Yutian Han, Jiang Zhu

**Affiliations:** ^1^ Department of Ultrasound, Women’s Hospital, Zhejiang University School of Medicine, Hangzhou, China; ^2^ Department of Ultrasound, Jinhua Hospital of Zhejiang University, Jinhua, China

**Keywords:** fetal tumor, head and neck, ultrasound, hemangioma, radiofrequency ablation

## Abstract

Large fetal head and neck tumors are being increasingly identified during prenatal examination and tend to have a poor prognosis. Nevertheless, appropriate intrauterine interventions at suitable periods can improve pregnancy outcome. Ultrasound-guided puncture biopsy of the solid fetal head and neck mass and radiofrequency ablation of a portion of the tissue can clarify the tumor pathology and reduce the tumor size, respectively. These treatment methods are reproducible and associated with reduced trauma and complications.

## Introduction

Fetal tumors are relatively rare, with a reported incidence of 1.7–13.5 per 100,000 live births (1). However, the true incidence of these tumors, which were also found in approximately 0.5% of stillbirths and 1.2% of fetuses with birth defects (2, 3), is often underestimated. Among them, fetal head and neck tumors account for a substantial proportion, with the most common histopathological types being hemangioma and lymphangioma (4). According to existing reports, most head and neck tumors are benign lesions; nevertheless, the prognosis of a few fetuses generally remains poor (5, 6). Abnormal developmental metabolic requirements due to excessive tumor growth and unfavorable tumor location often affect fetal development and may lead to several serious complications, such as airway obstruction, fetal edema, cardiac insufficiency, hydramnios, or even death (7, 8). Although most fetal head and neck tumors or malformations can be treated after birth, conditions of some fetuses with tumors deteriorate rapidly during gestation. In such cases, passive waiting for postpartum treatment may lead to the fetus missing the best treatment opportunity, resulting in a poor prognosis. The perinatal survival rate and quality of life can be improved if appropriate intrauterine treatment is offered before the damage caused is irreversible (9–11). We recently conducted the first biopsy and treatment and achieved good results.

## Case report

**Figure 1:**
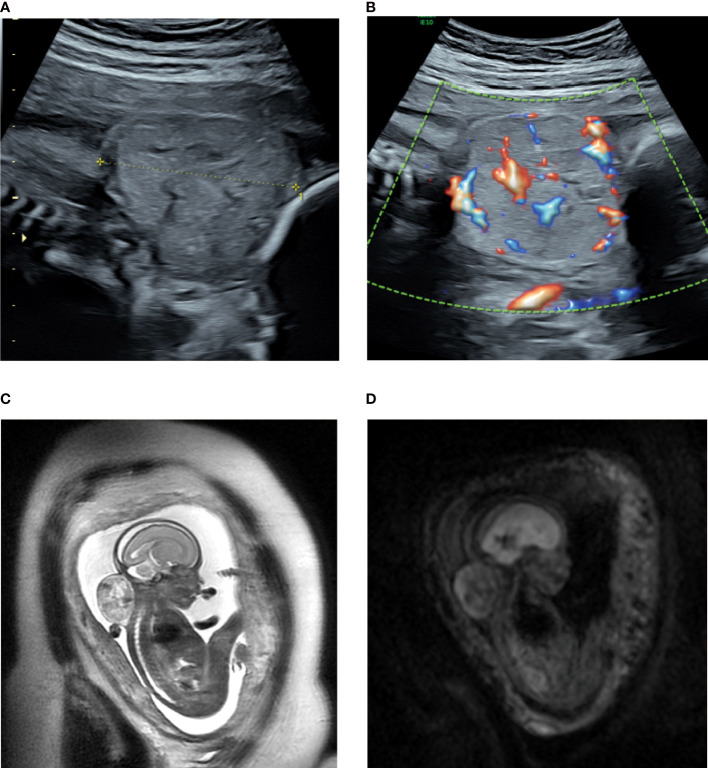
**(A, B)** Sonogram of the fetus with a posterior neck tumor at 23 weeks of gestation. **(A)** sagittal section; **(B)** color Doppler flow imaging; **(C, D)** Magnetic resonance multiple sequence imaging of the fetal neck tumor at 23 weeks of gestation. **(C)** sag T2SSFSE; **(D)**: Ax DWI b = 600.

A 30-year-old pregnant woman, gravida 3, para 0 (G_3_P_0_000), was first diagnosed at 23 weeks of gestation as having a solid tumor at the posterior aspect of the fetal neck through fetal ultrasound examination. No significant abnormalities were observed in the previous first-trimester fetal ultrasound scans, and nuchal translucency was 2.5 mm. Noninvasive prenatal testing indicated a low risk, thereby ruling out fetal chromosomal abnormalities. Two-dimensional ultrasonography revealed a solid, heterogeneous echogenic exophytic mass of approximately 4.2 × 3.7 × 4.0 cm that was well demarcated from the spine, and the leading edge was close to the skin (**Figure 1A**). Color Doppler sonography revealed slightly abundant arterial and venous blood flow signals at the bottom near the spine and a slight blood flow signal inside (**Figure 1B**). Fetal magnetic resonance imaging, performed the following day, confirmed the presence of this tumor with high–low mixed signals and a clear boundary on T2-weighted imaging. In addition, no airway extension or obstruction was observed (**Figures 1C, D**). The radiologist first considered the tumor as a teratoma or hemangioma but could not make a definitive diagnosis. The tumor volume increased rapidly within 6 weeks to approximately 6.0 × 5.6 × 5.5 cm at 29 weeks of gestation, which represents an approximately three-fold increase in size. Fetal echocardiography indicated that the fetal cardiothoracic transverse diameter ratio significantly increased to 0.67. In addition, slight tricuspid regurgitation was noted. At this time, fetal biometry and the amniotic fluid volume were normal, and no obvious signs of placentomegaly, ascites, pleural effusions, or integumentary edema were noted. If the fetal neck tumor continued to grow without receiving appropriate intervention in a timely manner, the large tumor may cause fetal hyperflexion, affecting the development of the cervical spine and the rest of the spinal cord and compressing the blood vessels or airway in the neck, leading to more severe complications. Moreover, arteriovenous shunts in the tumor could cause high-output cardiac failure in the fetus. The pregnant woman recently perceived a marked decrease in fetal movement at 28-29 weeks of gestation. Therefore, after extensive counseling about all options, including fetal open surgery, fetoscopic surgery, radiofrequency (RF) ablation, palliative care, and pregnancy termination, with experts from obstetrics, genetics, ultrasound, and pediatric surgery fields, the family was fully counseled about the risks and benefits of each treatment regimen. Eventually, RF ablation was attempted, and long-term follow-up was planned to determine the efficacy. As a benign or malignant diagnosis could not be definitively established with imaging, the fetal neck mass was first biopsied, followed by RF ablation.

To ensure the safety of the operation, *ex vivo* fresh liver tissue was used as a model for surgical rehearsal, and the power and time required for RF ablation were determined. An RF generator (Cool-tip RF system: Covidien, Bolder, USA/VIVA RF system:STARmed, Goyang, Korea) and an 18-gauge, 1.0cm active-tip internally cooled electrode (Well-Point RF Electrode: STARmed, Goyang, Korea) were used.An 18-gauge, 15-cm needle electrode was inserted and heated in the center of liver tissue under ultrasound guidance. Attempts were made to ablate the liver tissue with different levels of progressively increasing RF energy (10–100 W). In addition, a different ablation time was considered. During RF therapy, the high temperature generated around the needle carbonizes local tissues, resulting in increased impedance, which is the main factor affecting the ablation volume. When the RF energy was set to 50 W, the ablation sphere diameter was approximately 1.5 cm after 3 min (**Figure S1A**), and when the RF energy was 100 W, the diameter was approximately 2.5–3.0 cm in the same period (**Figure S1B**).

**Figure 2:**
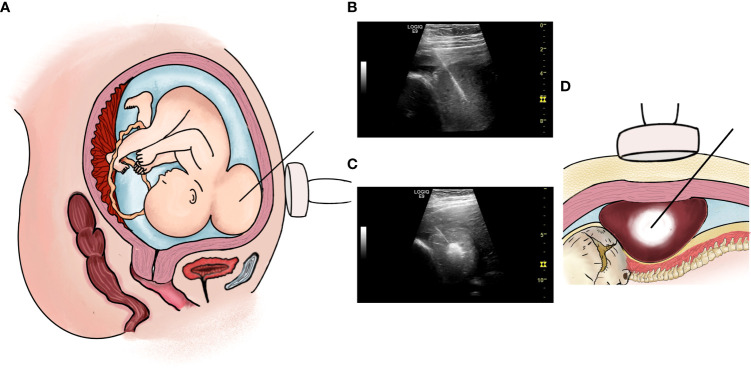
**(A, B)**: Percutaneous intrauterine biopsy at 29+ weeks of gestation. **(A)** Operation pattern diagram; **(B)** Percutaneous intrauterine biopsy under ultrasound guidance during operation. **(C, D)**: RF ablation therapy at 29+ weeks of gestation. **(C)** Operation pattern diagram; **(D)** RF ablation therapy under ultrasound guidance during operation.

The blood routine, blood biochemistry, thyroid function, and blood coagulation function of the pregnant woman were approximately normal, and no obvious contraindications were noted for surgery. The surgery was performed at 29 weeks of gestation in the operating room. Magnesium sulfate was intravenously infused preoperatively to reduce uterine sensitivity, and 1.5 g of cefuroxime sodium was administered as prophylaxis. Routine preoperative disinfection was performed. After local anesthesia in the supine position of the pregnant woman and intramuscular anesthesia of the fetus, the placenta and fetal vital organs were avoided, and the puncture point was determined under ultrasound guidance. A biopsy needle with an internal groove (length: 2.2 cm; Biopsy Needles, VPA18/10 18G × 10 cm, GALLINI S.R.L.) was inserted into the uterine cavity through the anterior abdominal wall of the pregnant woman, and the direction was adjusted to avoid the main vessels and then enter the tumor from the posterior neck of the fetus. Two tumor tissue biopsy samples were fixed with formalin and sent for pathological examination (**Figures 2A, B**). RF ablation was performed immediately after biopsy by inserting the RF electrode in the center of the tumor, 1.5–2.0 cm away from the edge of the tumor tissue (**Figures 2C, D**). The total ablation time was 8 min, including 2 min for 50 W and 6 min for 100 W, with a total ablation range of approximately 2.8 × 1.6 × 3.1 cm. Almost no obvious bleeding was noted at the puncture and ablation sites during the operation, and color Doppler flow imaging revealed a marked decrease in blood flow signals of the tumor after ablation. The vital signs of the pregnant woman were stable and she experienced little discomfort. The fetal heart rate was normal throughout the operation. Postoperatively, we paid close attention to contractions, abdominal pain, and vaginal bleeding.

**Figure 3:**
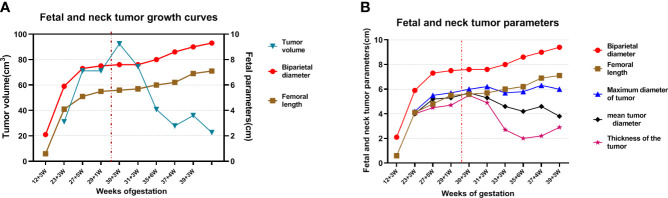
Fetal and neck tumor growth curves. “… in red” indicates the time that the fetus received intrauterine treatment. **(A)** The red curve: biparietal diameter; brown curve: fetal length; blue curve: tumor volume; **(B)** red curve: biparietal diameter; brown curve: fetal length; blue curve: maximum tumor diameter; black curve: mean tumor diameter; purple curve: tumor thickness.

**Figure 4:**
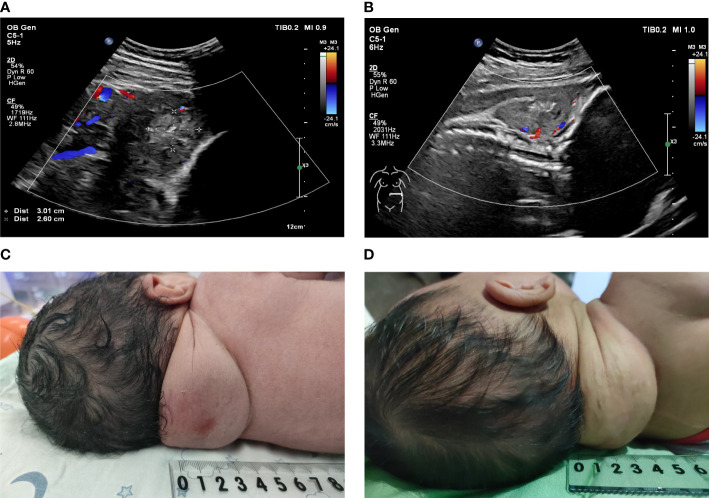
**(A, B)**: Doppler ultrasonography was used to observe the blood flow signal of the fetal posterior neck tumor after surgery. **(A)** One week after surgery; **(B)** One month after surgery. **(C)**: A solid and soft mass of approximately 6 × 5 × 4 cm protruded from the posterior neck of the newborn. **(D)**: A solid and soft mass of approximately 5 × 3 × 3 cm protruded from the posterior neck of the 42-day-old infant.

The fetal posterior neck solid tumor was identified as a congenital hemangioma (CH) through subsequent biopsy and histopathology (**Figure S2**). Therefore, the pregnant woman was closely followed after discharge. The first follow-up was conducted 1 week after surgery, and subsequent follow-ups were conducted once every 2 weeks. Follow-up ultrasound examination from immediately after operation to 33 weeks of gestation revealed that the tumor volume and the blood flow signal of the neck tumor were both significantly reduced (**Figures 4A, B**). The thickness of the tumor was measured in the sagittal section of the fetus, which was significantly decreased, whereas the reductions in the maximum and mean tumor diameters were not as obvious as that in the thickness (**Figure 3B**). In addition, the pregnant woman felt the fetus movement was becoming regular, improving significantly compared with almost no movement before surgery. Subsequently, no significant abnormality was observed in fetal growth parameters. The cardiothoracic ratio became normal (approximately 0.34–0.45), and tricuspid regurgitation was no longer observed. However, during follow-up, the fetal tumor size slightly increased to 6.3 ×5.2 × 2.2 cm at 37 weeks of gestation.

At 39 + 3 weeks of gestation, the pregnant woman was admitted due to premature rupture of membranes. Considering that vaginal delivery may lead to fetal dystocia, tumor rupture, and fetal distress caused by a compression of the birth canal all due to the presence of a fetal neck tumor, clinicians performed a cesarean section. A male newborn weighing approximately 3,360 g was delivered with an Apgar score of 10; the newborn took spontaneous breaths immediately. A fairly soft, bruised mass measuring approximately 6 × 5 × 4 cm was seen protruding from the posterior neck of the newborn. Pigmentation of approximately 1.0 × 1.0 cm was visible at the puncture point, and no obvious scars or burn marks were observed (**Figure 4C**). The spine curvature of the newborn was nearly normal,while the neck tumor did not affect the supine or lateral position of the fetus. The newborn had no obvious anemia or coagulation dysfunction. Considering that postpartum CH in the neck was mostly rapidly involuting CH (RICH), the pediatricians recommended close follow-up to monitor tumor growth and the growth and development of the child rather than immediate surgical resection. In the 42-day-old infant, the posterior neck mass had shrunk (5 × 3 × 3 cm) and the skin on the package surface was very loose (**Figure 4D**). The infant’s growth and development indicators were good. Serial monitoring of the infant is planned for the future.

## Discussion

CH is the most common perinatal hemangioma, mostly being single and occurring in the fetal head and neck. It is a benign endothelial tumor that completes the proliferative phase *in utero* and often stops growing after delivery (1, 12). CH is very rare, which makes accurate determination of its incidence difficult. CH can be divided into three types: RICH, partially involuting CH, and noninvoluting CH. The neck lesions are mostly RICH and conservative treatment is often adopted. Nevertheless, many complications are still observed in fetuses with CH, which are mainly related to tumor location and size. The tumor in the anterior neck may lead to abnormal lung development of the fetus because of airway obstruction (7, 8). Esophageal compression can affect the swallowing function in the fetus, resulting in hydramnios (7, 8). Large hemangiomas can also cause serious complications, such as high-output heart failure, severe anemia, and even fatality by the excessive flow of blood into the large hemangioma (8, 13). These are closely related to the prognosis of the fetus. Hence, accurate prenatal diagnosis and risk assessment of complications are critical in determining whether prenatal interventions should be performed.

In our case, the fetal neck tumor grew rapidly and a trend of further increase was noted during follow-up. However, the fetal growth and development indicators declined, as illustrated through the biparietal diameter and femoral length at 27–29 weeks of gestation, which suggested a trend of intrauterine developmental delay (**Figures 3A, B**). At the same time, the fetus showed a tendency of early heart failure. If we did not intervene in time, the fetus may have developed severe complications. Combined with relevant literature reports, intrauterine interventions after fetal edema are often associated with a poor prognosis and a high fetal mortality rate (8, 10, 11).After careful discussions among members of our multidisciplinary team, timely intrauterine interventions were performed based on the medical level of our center and the family’s consent.

At present, effective fetal intrauterine therapies for the tumor have gradually been developed and applied clinically, including fetal open surgery (14), laser ablation, and RF ablation (9–11). Fetal open surgery is a difficult procedure with few indications that is associated with the incidence of premature birth, intrauterine death of fetuses, and complications in pregnant women (14), which limit the development of this procedure. With the advancement in fetal surgery, intrauterine therapy has gradually become minimally invasive. Laser and RF ablation can provide a safe and minimally invasive alternative for fetal tumor treatment. Laser ablation is mostly used to ablate superficial vessels with a small ablation range and the operation is typically of longer duration (11), whereas RFA used to treat liver tumors and uterine fibroids has become mature and has been applied in fetal intrauterine therapy in recent decades, and many successful cases of RF ablation of fetal sacrococcygeal teratomas have been reported abroad (9). Therefore, we opted for RF ablation.

The solid mass in the posterior neck of the fetus is radiographically difficult to distinguish from teratoma, congenital fibrosarcoma, lymphangioma, and other perinatal head and neck vascular tumors. Ultrasound-guided biopsy can clarify the pathological type at tumor diagnosis, relieve anxiety in pregnant women and their families, reduce the rate of fetal loss, and further guide our subsequent pregnancy management. RF ablation was performed immediately after the biopsy both to reduce the duration of the entire operation and to prevent bleeding at the puncture site. The objectives of surgery were to reduce the size and blood flow of the tumor, slow the growth rate of the tumor, improve the fetal intrauterine environment, enable the fetus to grow safely to term, and provide conditions for further treatment after delivery. These measures are comply with the basic principles of fetal therapy (15). Moreover, the associated risks are generally minor and acceptable to the mother and the family.

Intrauterine fetal tumor treatment is often associated with good outcomes when only local tissue is ablated, whereas the outcomes of ablation of a large range of tissues are often poor (9–11). In this case, to avoid burning the fetal skin and surrounding tissues and the serious side effects caused by a large one-time ablation range such as thromboembolism caused by microbubbles generated by thermal coagulation, hyperkalemia caused by massive tissue necrosis or hemorrhage, and hemolysis (9, 10), we ablated only a portion of the tumor center with a safety margin. Referring to the pre-test results and combining with the actual intraoperative ablation situation, we choose 50 and 100 W to ablate a part of the tumor tissue, with a total ablation time of 8 min. The intrauterine environment of the fetus has significantly improved after surgery. The tumor slightly increased in size in the third trimester, and the growth and development indices of the fetus continued to increase (**Figure 3**). No indication was noted for a repeat ablation.

These fetal interventions also have limitations. First, the fetal tumor should be sufficiently large to avoid damage to the surrounding tissue. Second, it should be ensured that no placenta and vital fetal organs are present between the tumor and abdominal wall. More importantly, the operator must have a rich experience in RF ablation.

In conclusion, intrauterine puncture biopsy clarifying the tumor type pioneers a new approach that is essential for prenatal diagnosis and management. Ultrasound-guided percutaneous biopsy and RF ablation of solid fetal neck tumors represent a substantial advancement in the surgical treatment of fetal neck tumors. These treatment methods are associated with less trauma and fewer complications and allow repeatable operations.

## Data availability statement

The original contributions presented in the study are included in the article/**Supplementary Material**. Further inquiries can be directed to the corresponding author.

## Ethics statement

The study was approved by the ethics review board of Department of Ultrasound, Women’s Hospital, Zhejiang University School of Medicine (No. 120212147) in accordance with the Declaration of Helsinki. Written informed consent to participate in this study was provided by the participants’ legal guardian/next of kin. Written informed consent was obtained from the individual(s), and minor(s)’ legal guardian/next of kin, for the publication of any potentially identifiable images or data included in this article.

## Author contributions

Guarantors of integrity of entire study, JZ. Study concepts/study design or data acquisition or data analysis/interpretation, JZ, SSL, QL. Manuscript drafting or manuscript revision for important intellectual content, JZ,SL,QL, XFF, MYW. Approval of final version of submitted manuscript, JZ, QL, SSL. Agrees to ensure any questions related to the work are appropriately resolved, all authors. Literature research, JZ, SSL, QL, XS, HQD, GJK, XNL, YTH. clinical studies, JZ, SSL, QL, QGZ, LCW. Manuscript editing, all authors. All authors contributed to the article and approved the submitted version.

## Funding

This work was supported by the National Natural Science Foundation of China (grant numbers: 81974470 to JZ ) and the Natural Science Foundation of Zhejiang Province of China (LY18H180001 and LZ22H180001 to JZ ).

## Conflict of interest

The authors declare that the research was conducted in the absence of any commercial or financial relationships that could be construed as a potential conflict of interest.

## Publisher’s note

All claims expressed in this article are solely those of the authors and do not necessarily represent those of their affiliated organizations, or those of the publisher, the editors and the reviewers. Any product that may be evaluated in this article, or claim that may be made by its manufacturer, is not guaranteed or endorsed by the publisher.
