# Supplementary material for: Case report: Ultrasound-guided intrauterine biopsy and RF ablation therapy for fetal posterior neck solid tumor: first successful report
Source: Front Oncol. 2022 Aug 23;12:913694. doi: 10.3389/fonc.2022.913694 (PMC9446077; doi:10.3389/fonc.2022.913694)
Supplement: Supplementary file 1 [file DataSheet_1.docx]

Supplementary Material


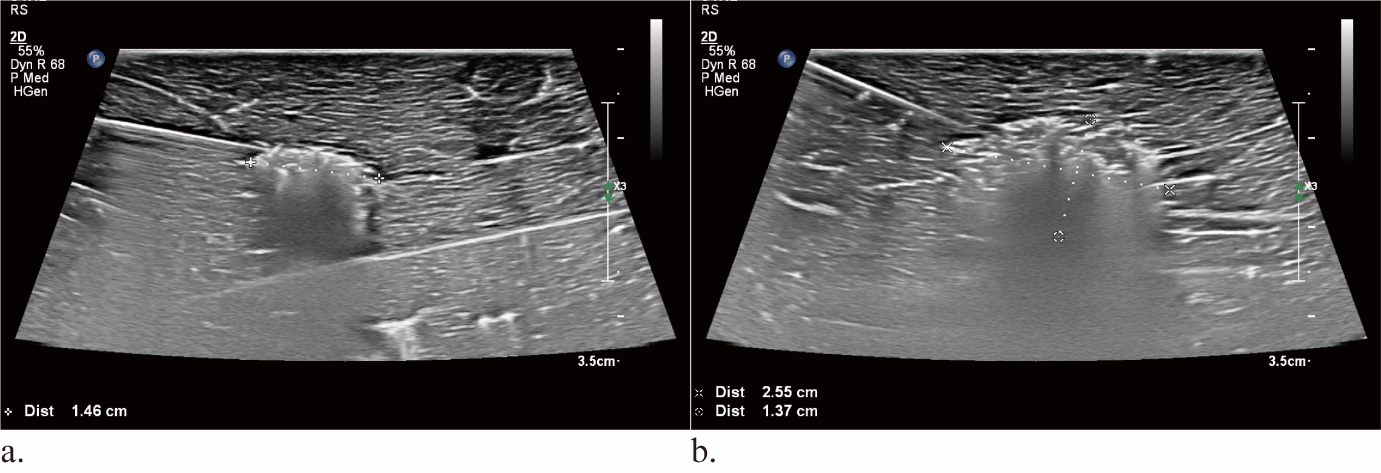


**Supplementary Figure 1.** a-b: Liver tissue for operation rehearsal 3 minutes after ablation. a. RF energy: 50 W; b. RF energy: 100 W.


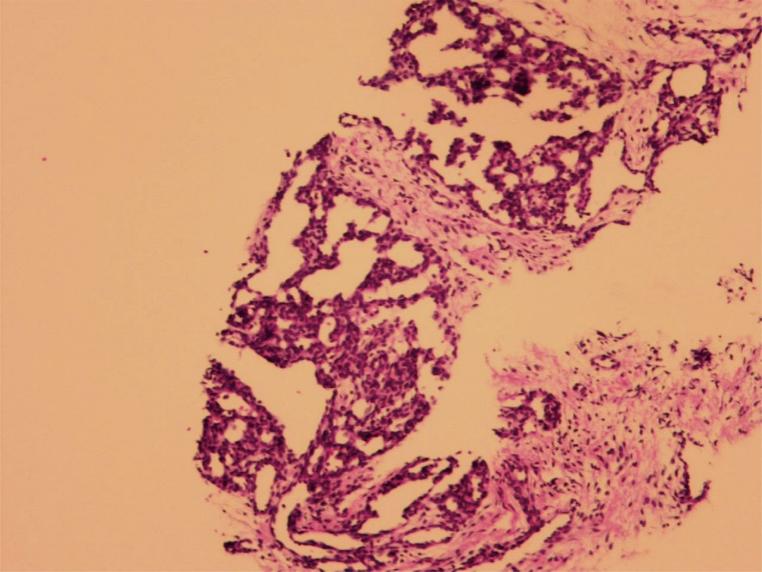


**Supplementary Figure 2.** Biopsy indicated congenital hemangioma: Hematoxylin - eosin staining was positive[Original magnification:100×]
